# Analytical Solution for the Potential Distribution in the Channel of A Graphene Field-Effect Transistor Validated with a Custom-Fabricated Test Platform

**DOI:** 10.1021/acsaelm.6c00360

**Published:** 2026-04-30

**Authors:** Antonio Cantudo, Francisco Pasadas, Anibal Pacheco-Sánchez, Rem Elnahas, Miguel Muñoz Rojo, Juan Bautista Roldán

**Affiliations:** † Departamento de Electrónica y Tecnología de Computadores, Facultad de Ciencias, 16741 Universidad de Granada, 18071 Granada, Spain; ‡ Pervasive Electronics Advanced Research Laboratory (PEARL), Facultad de Ciencias, Universidad de Granada, 18071 Granada, Spain; § Catalytic Processes and Materials Group, Faculty of Science and Technology, MESA+ Institute for Nanotechnology, University of Twente, P.O. Box 217, 7500 AE Enschede, The Netherlands; ∥ Thermal Engineering Group, Faculty of Engineering Technology, University of Twente, P.O. Box 217, 7500 AE Enschede, The Netherlands; ⊥ Institute “Carlos I” for Theoretical and Computational Physics, University of Granada, 18071 Granada, Spain

**Keywords:** GFET, analytical model, potential distribution, graphene, in-channel potential, transistor, circuit simulation

## Abstract

A comprehensive analytical framework for computing the potential distribution in graphene-based field-effect transistors (GFETs) is presented. The work introduces a pioneering set of self-consistent and explicit closed-form expressions that enable an accurate description of the internal potential profiles along the graphene channel. The proposed models are experimentally validated using a custom-fabricated test platform based on global back-gated GFETs incorporating two in-channel terminals, which allow direct measurement of the local potential drop at different channel positions and provide unique experimental access to the internal electrostatics of the device. Experimental drain current characteristics and in-channel potential measurements are systematically compared with model predictions, showing excellent agreement over a wide range of bias conditions and validating the analytical framework in both unipolar and ambipolar operating regimes. The extracted potential distributions enable a clear physical interpretation of key transport phenomena in GFETs, including the onset of channel pinch-off associated with maximum transconductance and the transition between unipolar and ambipolar transport regimes. Owing to their analytical nature and computational efficiency, the proposed pioneering models provide a powerful tool for rapid device evaluation, optimization, and interpretation, with direct relevance to graphene-based analog and radio frequency electronics, sensing, flexible electronics, and thermoelectric applications.

## Graphical abstract

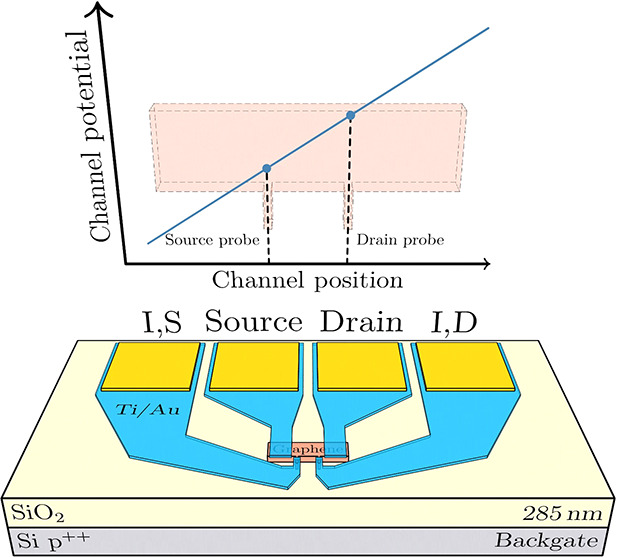

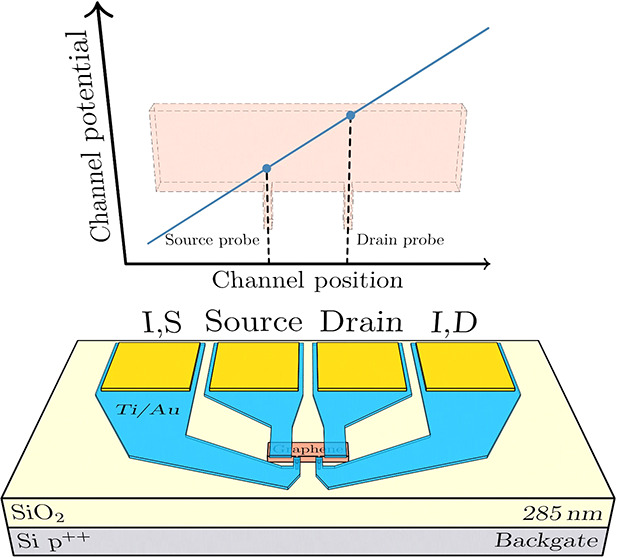

## Introduction

Graphene-based field-effect transistors (GFETs) have attracted considerable attention in recent years due to the unique electronic properties of graphene that are physically rooted to the massless-Dirac Fermions nature of the carriers on its distinctive band-structure.^1,2^ These properties include extremely high carrier mobility, ambipolar transport, excellent conductivity, and the ability to sustain high current densities.^3,4^ These characteristics position GFETs as promising candidates for high-frequency electronics,^5–8^ radio frequency circuits,^9^ flexible and transparent devices,^10,11^ thermal effects characterization,^12^ and highly sensitive chemical and biological sensors.^13^

The distribution of electrostatic potential along a transistor channel is a fundamental physical metric^14,15^ that influences key characteristics such as the quasi-current saturation,^16^ short-channel effects,^17^ carrier injection from contacts,^18^ and the intrinsic ambipolar behavior of graphene. In conventional Si MOSFETs, analytical models of the channel potential have been instrumental in providing deep insight into device physics, enabling rapid exploration of design spaces, guiding experimental optimization, and serving as the foundation for compact circuit models.^14,15^ Although several compact models for GFETs have been reported in the literature,^19–24^ these approaches typically evaluate carrier transport between the channel boundary conditions imposed at the source and drain. As a consequence, the local electrostatic behavior and the spatial distribution of the electrostatic potential within the interior of the channel cannot be explicitly resolved, limiting physical insight into the internal carrier transport mechanisms. This lack of a detailed, position-dependent electrostatic description remains a major challenge for GFETs, where the local potential profile governs the carrier concentration along the channel and directly impacts the device performance.

In contrast, GFET studies have largely relied on numerical simulations, including finite-element and finite-difference methods, drift-diffusion (DD) self-consistent solvers, and technology computer-aided design tools, to estimate channel potentials and current–voltage behavior.^25–29^ While these numerical approaches provide an accurate position-dependent electrostatic description, they are computationally intensive and offer limited flexibility for extensive parametric studies, optimization, and integration into circuit-level simulations.

In this context, the development of an analytical model describing the potential distribution along a GFET channel represents a significant advancement. There is currently no comprehensive explicit analytical model reported in the literature that captures the potential distribution from source to drain in a GFET, while taking into account the intrinsic properties of graphene, such as its linear density of states and ambipolar charge transport. Such a model allows a direct and explicit connection between the physical and geometrical parameters of the transistor, such as gate voltage, channel length, contact resistance, dielectric environment, and the resulting electrostatic landscape within the channel.^30,31^ An analytical expression for the potential distribution also enables rapid evaluation of different geometries, operating conditions, and material properties without the need for extensive numerical simulations. The model can also serve as a versatile tool for investigating the interplay between electrostatics and carrier transport in graphene systems, providing benchmarks for more sophisticated numerical approaches and offering a framework for understanding deviations from ideal behavior observed in experimental devices. Additionally, the ability to predict the potential distribution analytically has direct implications for emerging applications such as graphene-based sensors, where the local potential governs the sensitivity and selectivity of the device to chemical or biological analytes,^32,33^ and flexible electronics, where the interplay between mechanical strain, electrostatics, and transport must be captured efficiently,^34^ including thermal effects.^35^

In this work, we present a fully analytical model of potential distribution along the channel of a GFET. The model is derived from fundamental-physics-based principles of electrostatics and carrier transport in graphene described by DD theory, accounting for the unique physical characteristics of the material and the device architecture. We demonstrate that the resulting expressions provide explicit relationships between the channel potential and key device parameters, enabling rapid evaluation, optimization, and interpretation of the experimental results. The model has been validated by using measurement data obtained from a complete characterization of GFETs with different channel lengths and geometrical configurations, enabling direct measurement of the potential at various positions along the device channel.

The paper is organized as follows. Experimental Section describes the device fabrication process. Device Characterization presents the characterization methodology. In Analytical Solution of the Potential Distribution Along the Graphene-Based Field-Effect Transistor Channel, the analytical solution for the potential distribution along the GFET channel is derived. Results and Discussion discusses the results obtained, and finally, Conclusions summarizes the main outcomes.

## Experimental Section

The device fabrication has been entirely conducted by Applied Nanolayers company (Netherlands). In particular, the monolayer graphene has been synthesized by chemical vapor deposition and later transferred on p-doped silicon wafers (ρ = 0.001–0.005 Ω cm) with 285 nm dry chlorinated thermal SiO_2_ (Nova Ltd.). Channel patterning has been performed through e-beam lithography and O_2_ plasma, while electrode patterning has been conducted through photolithography. E-beam metal evaporation has been adopted to deposit Ti/Au electrodes of, respectively, 5 nm and 50 nm thicknesses. All devices were capped with 40 nm Al_2_O_3_ by atomic layer deposition following the procedure described in ref 36, to protect the graphene layer from air pollutants and other absorbates. Al_2_O_3_ was removed through etching in defined regions of the electrode pads to ease probe access. Figure 1 shows a schematic of the fabricated devices along with SEM images of the designed graphene-based structures.

**Figure 1:**
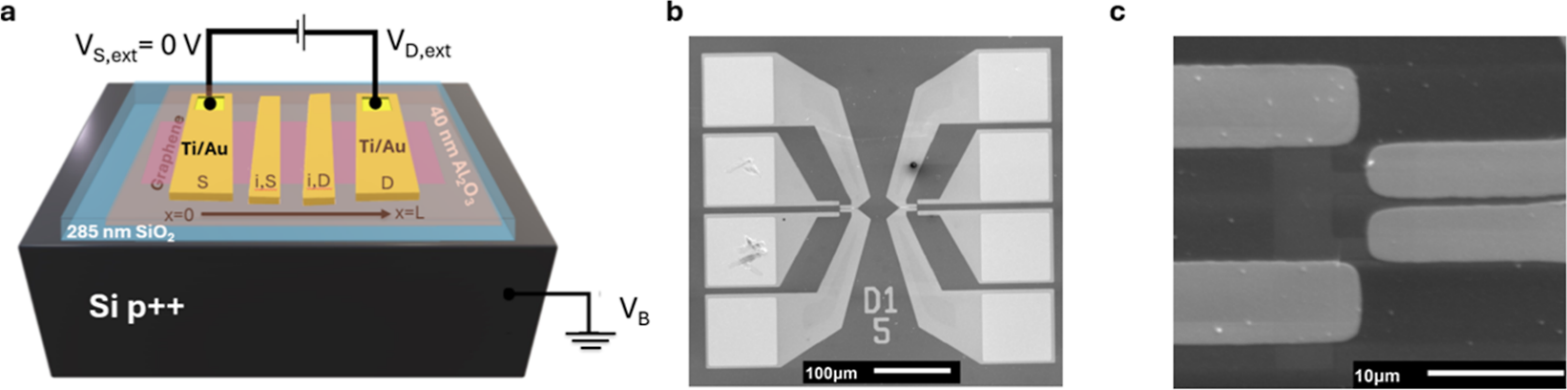
(a) Schematic of the fabricated global back-gated graphene field-effect transistors, (b) SEM images of the four-terminal structures, (c) zoomed in SEM image of the structures highlighting the four electrodes contacting the graphene channel.

For device operation, the drain (D) and source (S) terminals were utilized for the input bias, and the inner terminals (i,D and i,S, close to the drain and source channel edges, respectively) were used for the channel potential detection (cf. Figure 1a). To identify the electrical properties of the graphene channel, the “D” terminal was biased (and “S” was grounded), and the current flowing through the whole channel was monitored while the gate field was varied using the global back-gate electrode. Figure 1b,c shows the four terminals contacting the graphene channel in the fabricated GFETs. An additional SEM image of the four-terminal GFET structures is provided in Figure S1 in the Supporting Information. This configuration allows the potential drop to be measured at different positions along the channel. Similar structures, relying on a single inner electrode, were previously fabricated in ref 37, exploiting the potential distribution in the GFET channel for rectification and inversion purposes.

GFET test structures with different channel widths (from 5 μm to 50 μm) have been fabricated for contact resistance extraction in the same chip as the four-terminal devices. For each GFET type with a specific channel width, devices with the following channel lengths have been produced: 5 μm, 10 μm, 15 μm, 20 μm and 50 μm. SEM images of GFETs with different footprints are shown in Figure S2.

## Device Characterization

The graphene-based devices were characterized by using a Karl Suss probe station connected to a Keysight B1500A semiconductor parameter analyzer. For the transfer function characterization (*I*_DS_–*V*_B_), the external drain to source voltage (*V*_D,ext_) was set to 50 mV, while the gate voltage (*V*_B_) was swept between −60 and 60 V in 1 V steps, measuring the drain current on each step. A fairly high integration time of approximately 17 ms from a programmable logic controller connected to the semiconductor analyzer was used to minimize the effects of charge trapping.

### Contact Resistance Extraction

Conventional methodologies for contact resistance (*R*_c_) extraction applied in silicon devices, e.g., transfer length method, are not entirely suited for emerging transistors due to the different physics governing the device charge transport in these technologies.^38,39^ In this work, the device contact resistances have been obtained by following a GFET-suitable methodology described elsewhere^40^ and summarized as follows. By considering that the unipolar GFET drain current can be described by a first-order mobility degradation approach,^41^ the resistance *R*_c_, which includes the contributions of both the source-side and drain-side contact resistances, is extracted from the relation between the transconductance parameter (β) and the mobility degradation factor (θ), obtained from the transfer characteristics at a single external drain bias *V*_DS,ext_ from a set of devices with different channel lengths fabricated within the same technology. The former parameter (β) is obtained from the plot of the square of the Y-function^42^ (applied to the experimental data) versus the square of the overdrive extrinsic gate voltage *V*_BCO_ (=*V*_B_ – *V*_Dirac_ – *V*_D,ext_/2) (see Figure 2a), where *V*_Dirac_ is the back-gate bias yielding the minimum current. The Y function is defined as *Y* = *I*_DS_/*g*_m_^1/2^, where *g*_m_ (=d*I*_DS_/d*V*_B_) stands for transconductance. The parameter θ is obtained from the linear response of *R*_tot_ *Y* versus *Y* (see Figure 2b), where *R*_tot_ is the device total resistance. While Figure 2 presents the plots required for contact resistance extraction following the methodology proposed elsewhere^40^ for a GFET with a 10 μm wide channel, the corresponding results for a GFET with a 15 μm wide channel are shown in Figure S3.

**Figure 2:**
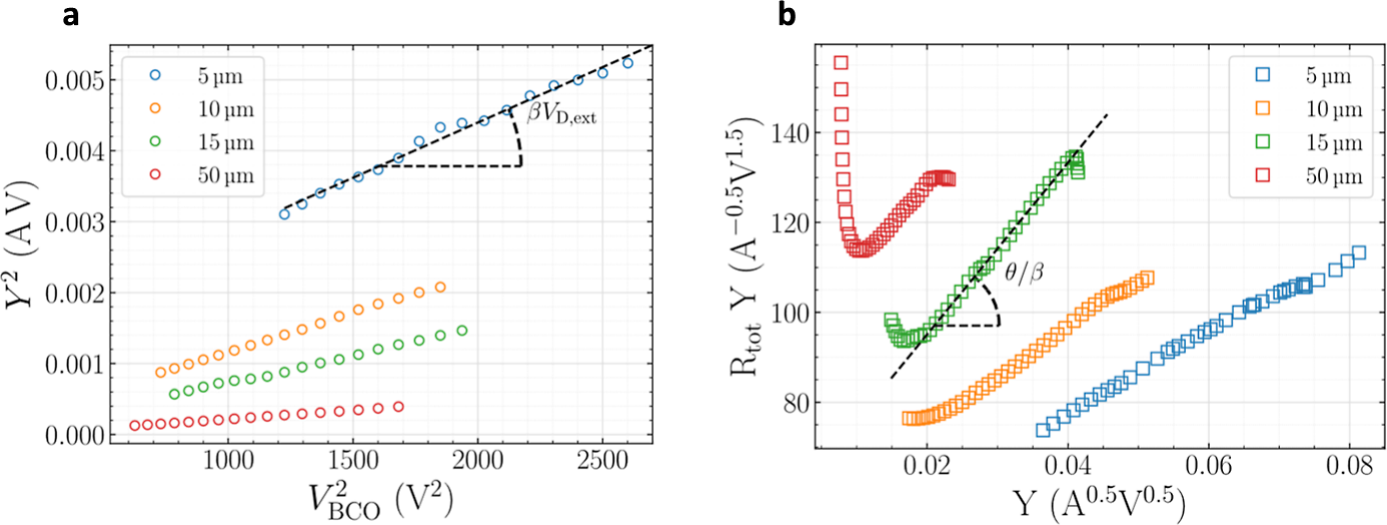
Plots required for contact resistance extraction using the methodology proposed in ref 40. (a) *Y* ^2^ versus *V*_BC0_^2^, and (b) *R*_tot_ *Y* versus *Y* for GFETs with 10 μm channel width and different channel lengths.

In order to validate the extracted values, they are introduced in the underlying *I*_DS,Y_ equation (Supporting Note 1), and the result is compared with the experimental data for all of the devices employed in the parameter extraction process. This comparison is presented in Figure 3, showing good agreement in the bias region where the extraction method was applied, i.e., within the GFET *n*-type operation regime. The agreement is consistent across devices with different channel lengths, as shown in Figure 3.

**Figure 3:**
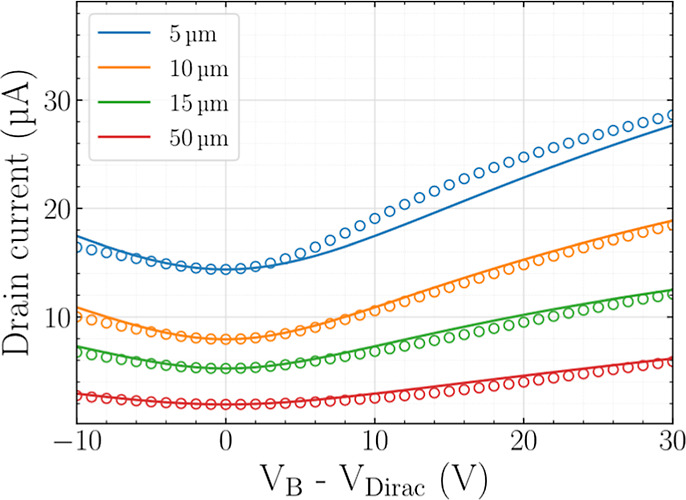
Measured (symbols) and modeled (solid lines, Supporting Note 1) transfer characteristics of GFETs with 10 μm channel width and different channel lengths (same fabrication technology).

The resulting *R*_C_ has first been extracted for each set of devices with different channel lengths but a common channel width (see Figure S4). Figure 4 presents the *R*_C_ values extracted for each channel width, highlighting its dependence on the device width.

**Figure 4:**
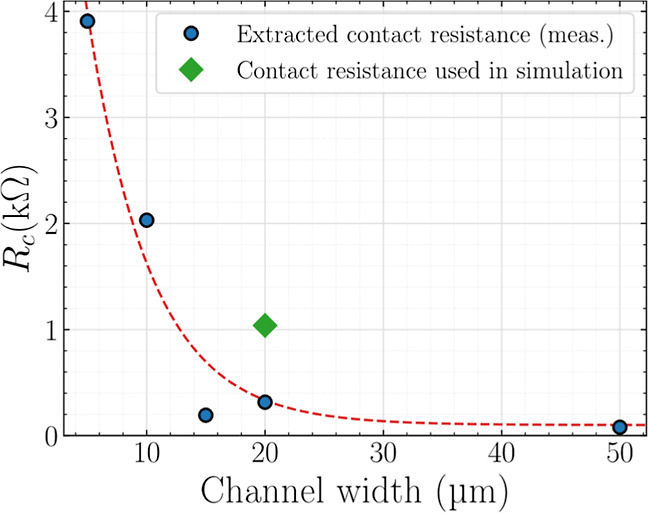
Contact resistance values (blue symbols) extracted with the adapted Y-function methodology for GFETs with different channel widths, and (green symbols) considered for physics-based modeling (cf. Results and Discussion). The red dashed line represents a polynomial fit and serves as a guide to the eye.

### Four Terminal Devices

Figure 5 illustrates the experimental setup employed for the electrical characterization of the four-terminal GFET device. Owing to intrinsic limitations of the parameter analyzer (Keithley 4200A), which does not allow simultaneous high-impedance voltage sensing at the in-channel electrodes, an external oscilloscope was used to measure the voltage at the inner channel terminals.

**Figure 5:**
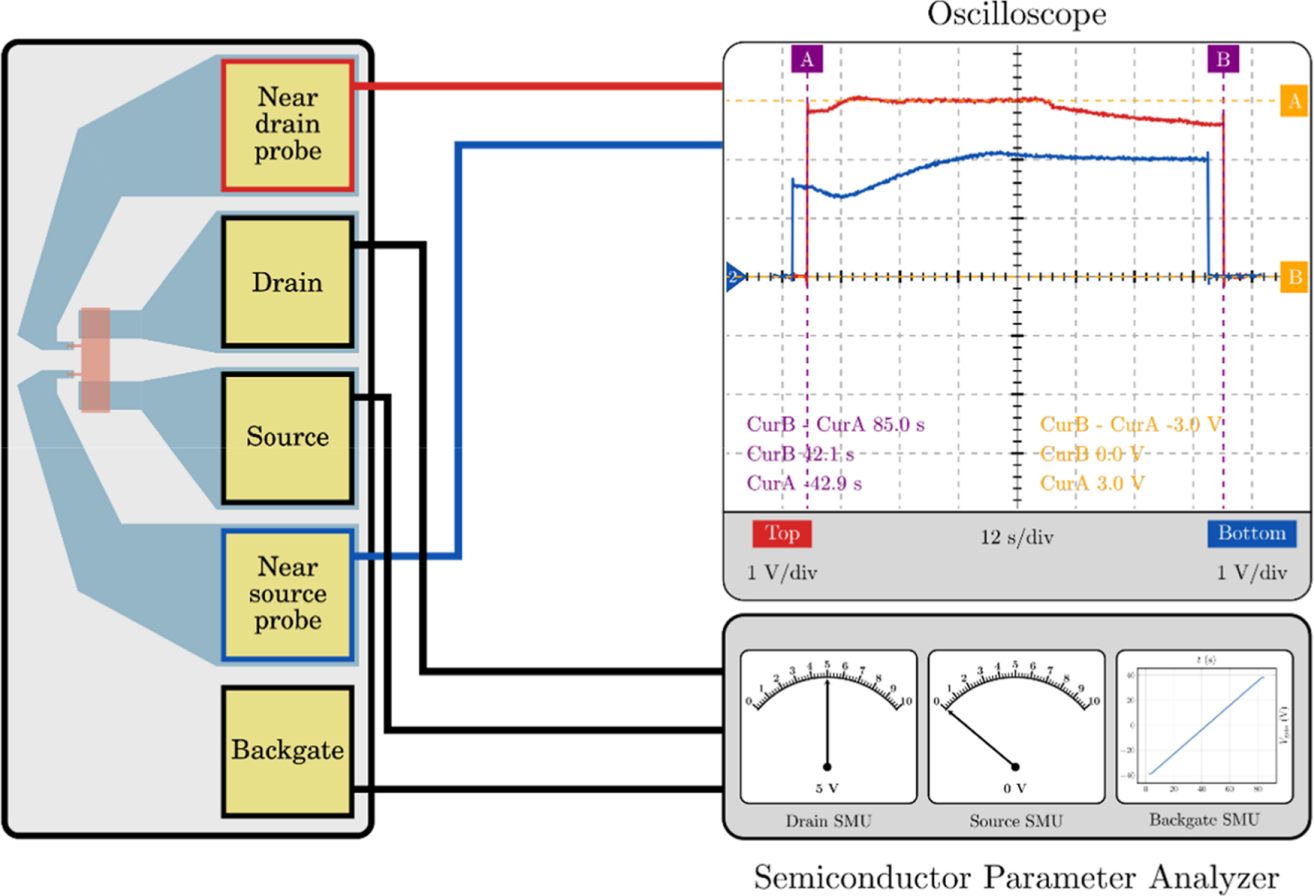
Experimental setup for the four terminal GFET characterization. The characterization procedure was conducted in two independent measurement steps: one involving the near drain probe (red curve), and a second involving the near source probe (blue curve). The semiconductor parameter analyzer is connected to the back-gate terminal, and source and drain pads, and the oscilloscope is connected to either the near drain or near source pads (in-channel electrodes).

The in-channel electrodes must be measured under high-impedance conditions to ensure that no significant current is drawn through the probing system, which could otherwise perturb the intrinsic drain-to-source transport characteristics of the GFET. For a drain-to-source bias of *V*_DS,ext_ = 5 V, the device exhibits a maximum channel resistance on the order of 10 kΩ. In this context, the oscilloscope input resistance of 1 MΩ is sufficiently large to guarantee negligible loading effects, thereby preventing measurement-induced artifacts.

The voltages at the inner electrodes located near the drain (*V*_i,D_) and the source (*V*_i,S_) were not recorded simultaneously. Consequently, the characterization procedure was conducted in two independent measurement steps: one involving the inner terminal located close to the drain (i,D; see Figure 1a and red curve in Figure 5), and a second involving the inner terminal located close to the source (i,S; see Figure 1a and blue curve in Figure 5). In each measurement step, the back-gate voltage (*V*_B_) was swept while both the GFET drain-to-source current were monitored using the parameter analyzer and the corresponding inner-terminal voltage was monitored using the oscilloscope. Throughout the entire experiment, the drain-to-source voltage was maintained at a *V*_DS,ext_ of 5 V.

Figure 6a presents the resulting transfer characteristics obtained from the two independent measurement steps, performed at different times, for a GFET with a channel width of 20 μm and a channel length of 30 μm. The inner electrodes were positioned between the longitudinal position (cf. Figure 1a) *x* ∼ 4–6 μm and *x* ∼ 24–26 μm along the channel. The layout of the device is provided in Figure S5 (Supporting Information). It can be observed that the two transfer curves in Figure 6a are nearly identical, thereby validating the proposed measurement approach. This result confirms that the oscilloscope input impedance is sufficiently high to avoid perturbing the device operation. Furthermore, the results demonstrate the excellent electrical stability of the fabricated GFETs as the *I*–*V* characteristics are reproducible across two independent measurements performed at different times.

**Figure 6:**
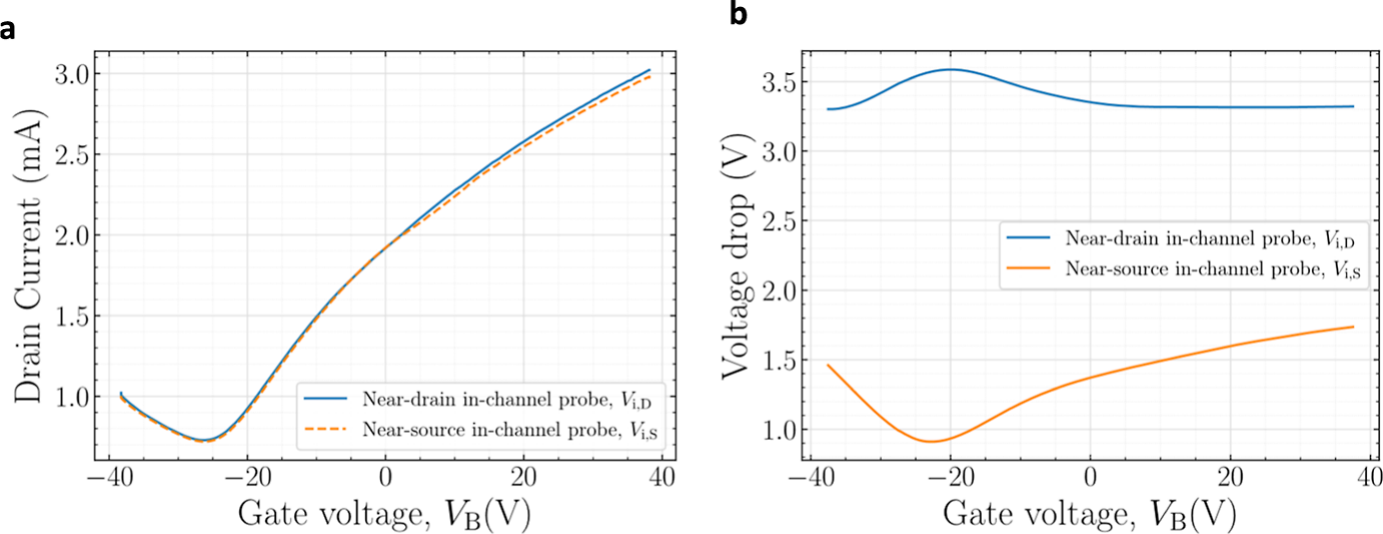
(a) Transfer characteristics and (b) in-channel voltage drop at *V*_DS,ext_ = 5 V of a GFET with a 20 μm wide and 30 μm long channel, measured in two independent steps performed at different times: one to monitor the potential drop at the in-channel electrode placed near the drain, and the other near the source.

## Analytical Solution of the Potential Distribution along the Graphene-Based Field-Effect Transistor Channel

The analytical solution of the channel potential is derived from the modeling framework developed by some of the authors elsewhere.^19,43^ The model assumes a transistor channel of width *W* and length *L*; the spatial coordinate *x* along the channel runs from 0 to *L*, as shown in Figure 1a. The electrostatic modulation of the carrier concentration in the graphene channel is modeled via a double-gate stack consisting of top- and back-gate dielectrics and the corresponding metal gates. The graphene sheet under electric control of the gate electrodes plays the role of the active channel. The model considers that the graphene channel is controlled by the gates from the drain and source edges; therefore, ungated areas such as access regions^28^ are not explicitly accounted for.

The graphene sheet is contacted at its edges by both the drain “D” and source “S” electrodes (cf. Figure 1a); carrier transport along the spatial coordinate *x* is produced between both terminals when a nonzero drain-to-source bias is applied, i.e., |*V*_D_–*V*_S_| > 0, where *V*_D_ (*V*_S_) is the drain (source) voltage. The 1D electrostatics of the GFET along a transversal cut can be obtained as follows^43^

1 $$Q_{net}\left(x\right)=-C_{t}\left(V_{G}-V_{G0}-V\left(x\right)+V_{c}\left(x\right)\right)-C_{b}\left(V_{B}-V_{B0}-V\left(x\right)+V_{c}\left(x\right)\right)$$

where *Q*_net_(*x*) = *q*[*p*(*x*) – *n*(*x*)] is the net sheet charge density; *q* is the elementary charge; *p*(*x*) and *n*(*x*) are the hole and electron carrier densities, respectively. *C*_t_ = ϵ_t_/*t*_t_ (*C*_b_ = ϵ_b_/*t*_b_) is the top- (back-) oxide capacitance per unit area, with ϵ_t_ (ϵ_b_) the top (back) dielectric constant and *t*_t_ (*t*_b_) the top- (back-) oxide thickness. The top (back) overdrive voltage is *V*_G_–*V*_G0_ (*V*_B_–*V*_B0_), where *V*_G_ (*V*_B_) is the top- (back-) gate potential, and *V*_G0_ (*V*_B0_) comprises the work-function difference between the top (back) gate and the graphene channel, as well as the effect of additional fixed charge owing to impurities or doping. Although the fabricated graphene devices employ a global back-gate configuration (Figure 1a), leading to *C*_t_ → 0 F in eq 1, the model is presented in its general form and includes the dual-gate configuration for completeness. Because of the presence of non-negligible contact resistances in GFETs, as addressed in Contact Resistance Extraction, the intrinsic source and drain potentials (*V*_S_ and *V*_D_ at the active channel edges) cannot be shorted to reference in practice;^25^ therefore, we adopted a general formulation where none of the terminals are grounded. Thus, the GFET is driven by terminal voltages defined with respect to some arbitrary reference point. The relation between external (*V*_D,ext,_ and *V*_S,ext_ = 0 V) and internal voltages (*V*_D_ and *V*_S_), assuming the contact resistances as lumped resistors at the drain (*R*_D_) and source (*R*_S_) metal–graphene interfaces, reads as

2 $$\begin{array}{l} V_{D}=V_{D,ext}-I_{DS}R_{D}\\ V_{S}=I_{DS}R_{S} \end{array}$$

where *V*_D,ext_ (*V*_S,ext_) is the externally applied bias to the drain (source) electrode, as shown in Figure 1a.

In eq 1, *V*(*x*) is the quasi-Fermi level along the graphene channel, i.e., the electrochemical potential, and it must fulfill the following boundary conditions: (i) *V*(*x* = 0) = *V*_S_ and (ii) *V*(*x* = *L*) = *V*_D_ at the source and drain edge-sides. *V*(*x*) is assumed to be the same for both electrons and holes because the generation/recombination times for carriers in graphene are very short (in order of ps);^44–46^ therefore, electron and hole quasi-Fermi levels do not deviate significantly from each other.^17,25^ *V*_c_(*x*) represents the position-dependent chemical potential, i.e., the shift of the Dirac potential with respect to the quasi-Fermi level. Electron and hole concentrations can be calculated as a function of *V*_c_(*x*) using the following equations, which are a result of the density of states of graphene (deduced from its linear dispersion relation), where the electronic states are occupied according to the Fermi–Dirac statistics^19^

3 $$\begin{array}{l} p\left(x\right)=\frac{\Delta^{2}}{2\pi {\left(\hbar v_{F}\right)}^{2}}+\frac{2}{\pi }{\left(\frac{k_{B}T}{\hbar v_{F}}\right)}^{2}F_{1}\left[\frac{qV_{c}\left(x\right)}{k_{B}T}\right]\\ n\left(x\right)=\frac{\Delta^{2}}{2\pi {\left(\hbar v_{F}\right)}^{2}}+\frac{2}{\pi }{\left(\frac{k_{B}T}{\hbar v_{F}}\right)}^{2}F_{1}\left[-\frac{qV_{c}\left(x\right)}{k_{B}T}\right] \end{array}$$

where Δ is the amplitude of the electrostatic potential inhomogeneity that causes the electron–hole puddles,^47^ $F_{1}$ is the Fermi–Dirac integral of first-order, *k*_B_ is the Boltzmann constant, *T* is the temperature, *ℏ* is the reduced Planck constant, $v_{F}=\sqrt{3}a\gamma_{0}/2\hbar$ is the Fermi velocity; where *a* = 2.49 Å is the graphene lattice constant^48^ and γ_0_ = 3.16 eV is the interlayer coupling.^49^ The term (*n*_res_/2 =) $\Delta^{2}/\left({2\pi \left(\hbar v_{F}\right)}^{2}\right)$ accounts for the contribution of the puddles to the residual carrier concentration. The net sheet charge density and the quantum capacitance of graphene, which is defined as *C*_q_(*x*) = ∂*Q*_net_/∂*V*_c_, result in ref 50

4 $$\begin{array}{l} Q_{net}\left(x\right)=\frac{2q{\left(k_{B}T\right)}^{2}}{\pi {\left(\hbar v_{F}\right)}^{2}}\left(F_{1}\left[\frac{qV_{c}\left(x\right)}{k_{B}T}\right]-F_{1}\left[-\frac{qV_{c}\left(x\right)}{k_{B}T}\right]\right)\\ C_{q}\left(x\right)=\frac{2q^{2}k_{B}T}{\pi {\left(\hbar v_{F}\right)}^{2}}\;\ln\left[2\left(1+\cosh\left[\frac{qV_{c}\left(x\right)}{k_{B}T}\right]\right)\right] \end{array}$$

*V*_c_(*x*) could be calculated as a function of the terminal biases (*V*_G_, *V*_B_, *V*_D_, *V*_S_) from eqs 1 and 4. However, this formulation is not convenient for an analytical solution. Using a square-root-based approximation for *C*_*q*_,^19,50^ it is possible to get an implicit expression for *V*_c_ that can be written in terms of elemental functions, which is more suitable for the analytical derivation as follows

5 $$\begin{array}{l} C_{q}\left(x\right)\approx kc_{1}\sqrt{1+{\left(V_{c}/c_{1}\right)}^{2}}\\ Q_{net}\left(x\right)=\int_{0}^{V_{c}}C_{q}\left(Ṽ\right)\;dṼ=\frac{kc_{1}}{2}\left(V_{c}\sqrt{1+{\left(V_{c}/c_{1}\right)}^{2}}+c_{1}\;\mathrm{asinh}\left[V_{c}/c_{1}\right]\right) \end{array}$$

where $k=2q^{3}/\left(\pi {\left(\hbar v_{F}\right)}^{2}\right)$ and *c*_1_=(*k*_B_ *T*/*q*) ln(4).

The charge transport is described following the DD theory, which implies that the carrier mean free path (MFP) is significantly shorter than the device length, *L*. The MFP is related to the graphene quality and values below 100 nm have been reported at room temperature.^47^ The GFET drain current, according to the DD theory, can then be evaluated as^43^

6 $$I_{DS}=qW\rho_{sh}\left(x\right)\mu \frac{dV\left(x\right)}{dx}$$

where ρ_sh_(*x*) = *p*(*x*) + *n*(*x*) is the transport carrier sheet density and μ is the effective mobility, considered the same for both types of carriers for the sake of obtaining an analytical description of the potential distribution, although the model could be extended for nonequal mobilities following the work of some of the authors.^51^ To maintain the model simplicity, the velocity saturation effect for the drift carrier velocity has not been considered; therefore, the model is limited to long-channel devices. Explicit expressions to account for saturation effects for the drift carrier velocity can be found in refs 19 and 43 .

Under the condition of symmetrical electron and hole mobilities, the transport carrier sheet density ρ_sh_(*x*) is approximated to its second-order Taylor expansion^50^

$$\rho_{sh}\left(x\right)\approx \frac{\Delta^{2}}{\pi {\left(\hbar v_{F}\right)}^{2}}+\frac{\pi {\left(k_{B}T\right)}^{2}}{3{\left(\hbar v_{F}\right)}^{2}}+\frac{q^{2}V_{c}^{2}\left(x\right)}{\pi {\left(\hbar v_{F}\right)}^{2}}$$ 7

The drain current equation must be integrated over the device length (*L*), and it is convenient to solve the integral by using *V*_c_ as the integration variable, consistently expressing ρ_sh_ as a function of *V*_c_. The drain current can thus be written as follows

8 $$I_{DS}=\mu \frac{W}{L}q\int_{V_{cs}}^{V_{cd}}\rho_{sh}\left(V_{c}\right)\frac{dV}{dV_{c}}\;dV_{c}$$

The chemical potentials at the source ${V_{cs}=V_{c}\left(x=0\right)|}_{V=V_{S}}$ and drain $V_{cd}={V_{c}\left(x=L\right)|}_{V=V_{D}}$ side-edges are the relevant quantities required to calculate the drain current. They can be straightforwardly determined by solving eq 1, where *Q*_net_(*x*) reads as eq 5. In addition, the quantity d*V*/d*V*_c_ can be derived from eq 1, and it reads as

9 $$\frac{dV}{dV_{c}}=1+\frac{C_{q}\left(V_{c}\right)}{C_{t}+C_{b}}$$

From eq 8, the following closed-form drain current equation is achieved^19^

10 $$I_{DS}=\frac{W\mu }{L}{\left\{\frac{k}{2}\left(\frac{kc_{1}V_{c}\left(c_{1}^{2}+2V_{c}+4c_{2}\right)\sqrt{1+{\left(V_{c}/c_{1}\right)}^{2}}}{8\left(C_{t}+C_{b}\right)}-\frac{kc_{1}^{2}\left(c_{1}^{2}-4c_{2}\right)\;\mathrm{asinh}\left[V_{c}/c_{1}\right]}{8\left(C_{t}+C_{b}\right)}+\frac{V_{c}^{3}}{3}+c_{2}V_{c}\right)\right\}}_{V_{cs}}^{V_{cd}}$$

where $c_{2}={\left({\pi k}_{B}T\right)}^{2}/3q^{2}+\Delta^{2}/q^{2}$ .

Since the drain current is the same at any point *x* in the channel (assuming there are no generation-recombination processes involved), we get from the DD transport model the following equation:

11 $$\begin{array}{l} x=\mu \frac{W}{I_{DS}}q\int_{V_{cs}}^{V_{c}}\rho_{sh}\left({Ṽ}_{c}\right)\left(1+\frac{C_{q}\left({Ṽ}_{c}\right)}{C_{t}+C_{b}}\right)\;d{Ṽ}_{c}\\ x=\mu \frac{W}{I_{DS}}{\left\{\frac{k}{2}\left(\frac{kc_{1}{Ṽ}_{c}\left(c_{1}^{2}+2{Ṽ}_{c}+4c_{2}\right)\sqrt{1+{\left({Ṽ}_{c}/c_{1}\right)}^{2}}}{8\left(C_{t}+C_{b}\right)}-\frac{kc_{1}^{2}\left(c_{1}^{2}-4c_{2}\right)\;\mathrm{asinh}\left[{Ṽ}_{c}/c_{1}\right]}{8\left(C_{t}+C_{b}\right)}+\frac{{Ṽ}_{c}^{3}}{3}+c_{2}{Ṽ}_{c}\right)\right\}}_{V_{cs}}^{V_{c}} \end{array}$$

### Methodology A—Self-Consistent Analytical Model of the Potential Distribution in a Graphene-Based Field-Effect Transistor Channel

The procedure to compute *V*(*x*) along the channel consists of the following steps:

(i) Determine the source and drain potentials: compute the source *V*_S_ = *V*(*x* = 0) and drain *V*_D_ = *V*(*x* = *L*) potentials dropped at the graphene channel edges using eq 2.
(ii) Self-consistent calculation of the chemical potentials at the channel edges. Evaluate the source and drain chemical potentials, ${V_{cs}=V_{c}\left(x=0\right)|}_{V=V_{S}}$ and $V_{cd}={V_{c}\left(x=L\right)|}_{V=V_{D}}$ , respectively, using eq 1 and the expression for *Q*_net_(*x*) in eq 5.
(iii) Determine the position-dependent channel potential. Calculate the position *x* of *V*_c_(*x*) from *V*_cs_ to *V*_cd_ using eq 11, by computing the drain current *I*_DS_ from the explicit expression in eq 10.
(iv) Compute the full potential distribution along the channel. Evaluate *V*(*x*) from source (*x* = 0) to drain (*x* = *L*) using eq 12, derived from eq 1:

12 $$V\left(x\right)=\frac{Q_{net}\left(x\right)}{C_{t}+C_{b}}+\frac{C_{t}}{C_{t}+C_{b}}\left(V_{G}-V_{G0}+V_{c}\left(x\right)\right)+\frac{C_{b}}{C_{t}+C_{b}}\left(V_{B}-V_{B0}+V_{c}\left(x\right)\right)$$

### Methodology B—Explicit Analytical Model of the Potential Distribution in a Graphene-Based Field-Effect Transistor Channel

Methodology A relies on a self-consistent solution, and therefore, it does not allow for a straightforward and direct evaluation of the impact of different geometries, operating conditions, or material properties on the potential distribution along the GFET channel. An explicit analytical expression for the chemical potential distribution (*V*_c_) enabling rapid evaluation has been proposed under the condition |*V*_c_| ≫ *k*_B_ *T*/*q*.^52^ This assumption mostly restricts its applicability to bias conditions in which the graphene channel exhibits unipolar conduction,^53^ that is, to operating regimes away from the Dirac point, excluding particularly gate biases near the Dirac voltage or low carrier density regimes. However, in particular, the calculation of the quantum capacitance and net sheet charge density in eq 5 can be simplified and approximated as *C*_q_ = *k*|*V*_c_| = Sign­[*V*_c_]*V*_c_ ^50^ and *Q*_net_ = *k*Sign­[*V*_c_]*V*_c_^2^/2, respectively.^52^

Under these conditions, steps (ii), (iii), and (iv) of Methodology A, which involve the calculation of the position-dependent potential distributions using eqs 5, 11, and 12, can instead be straightforwardly evaluated using eqs 13, 14, and 15, respectively:

13 $$V_{c}\left(x\right)=(\frac{1}{\pm k})\{\left(C_{t}+C_{b}\right)-\sqrt{{\left(C_{t}+C_{b}\right)}^{2}\pm 2k\left[C_{t}\left(V_{G}-V_{G0}-V\left(x\right)\right)+C_{b}\left(V_{B}-V_{B0}-V\left(x\right)\right)\right]}\}$$

where the positive sign applies when *C*_t_(*V*_G_ – *V*_G0_ – *V*(*x*)) + *C*_b_(*V*_B_ – *V*_B0_ – *V*(*x*)) < 0, and the negative sign otherwise.

14 $$x=L\frac{4\left(C_{t}+C_{b}\right)\left(-{V_{c}}^{3}\left(x\right)+{V_{cs}}^{3}-3c_{2}V_{c}\left(x\right)+3c_{2}V_{cs}\right)+3k\left(Sign\left[V_{cs}\right]{V_{cs}}^{2}\left({V_{cs}}^{2}+2c_{2}\right)-Sign\left[V_{c}\left(x\right)\right]{V_{c}}^{2}\left(x\right)\left({V_{c}}^{2}\left(x\right)+2c_{2}\right)\right)}{4\left(C_{t}+C_{b}\right)\left(-{V_{cd}}^{3}+{V_{cs}}^{3}-3c_{2}V_{cd}+3c_{2}V_{cs}\right)+3k\left(\mathrm{Sign}\left[V_{cs}\right]{V_{cs}}^{2}\left({V_{cs}}^{2}+2c_{2}\right)-Sign\left[V_{cd}\right]{V_{cd}}^{2}\left({V_{cd}}^{2}+2c_{2}\right)\right)}$$

15 $$V\left(x\right)=\frac{C_{t}}{C_{t}+C_{b}}\left(V_{G}-V_{G0}+V_{c}\left(x\right)\right)+\frac{C_{b}}{C_{t}+C_{b}}\left(V_{B}-V_{B0}+V_{c}\left(x\right)\right)+\frac{k\;\mathrm{Sign}\left[V_{c}\left(x\right)\right]{V_{c}}^{2}\left(x\right)}{2\left(C_{t}+C_{b}\right)}$$

Eqs 13–15 constitute a pioneering set of explicit closed-form expressions for calculating the channel and chemical potential distributions in GFETs. It is worth emphasizing that, since the potential distributions are governed purely by electrostatics, they are independent of the drain current and, consequently, of the carrier mobility and channel length when expressed as a function of the normalized coordinate *x*/*L*, as can be directly inferred from eqs 13–15. This implies that under the long-channel approximation, the potential profile exhibits a scaling invariance with respect to the channel length. However, this behavior no longer holds for short-channel devices, where two-dimensional electrostatic effects and carrier velocity saturation become significant. The impact of these effects on the spatial distribution of the channel potential has been numerically analyzed in ref 17. Furthermore, due to the non-negligible metal–graphene contact resistances, the intrinsic potentials at the source and drain edges of the graphene channel (*V*_S_ and *V*_D_, respectively) do depend on the drain current (and thus on the carrier mobility and channel length) as they are affected by the voltage drop across the contact resistances (cf. step (i), eq 2).

## Results and Discussion

As a preliminary verification step, we employed our in-house GFET computer-aided design (CAD) tool to perform a fitting of the current–voltage characteristics and to determine the potential distribution across the intrachannel electrodes. The testbench methodology involves cascading two GFETs with differing channel lengths to mimic the experimental setup shown in Figure 5. This approach is physically justified by the presence of the in-channel electrodes, which partition the graphene channel into contiguous regions that can be modeled as coupled GFET segments sharing the same global back gate. The intermediate node between two GFET segments represents the location of the in-channel contact, enabling direct access to the local channel potential. This configuration preserves current continuity and provides a circuit-level implementation of spatially distributed electrostatics along the channel. The corresponding circuit topology is depicted in Figure 7. This configuration enables the prediction of the voltage drop distribution by straightforwardly solving Kirchhoff’s laws using the circuit simulator Keysight Advanced Design System (ADS).

**Figure 7:**
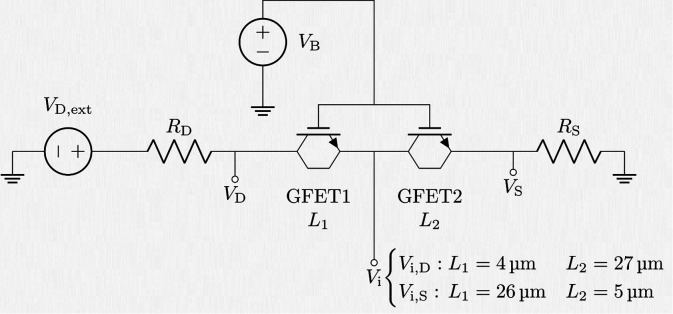
Implemented circuit topology used to mimic the experiments described in Contact Resistance Extraction. Two GFETs based on the technology described in Table 1 are connected in cascade. Metal–graphene contact resistances are included as lumped resistors. Two different channel-length configurations are employed: *L*_1_ = 4 μm and *L*_2_ = 27 μm to extract the intrinsic potential near the drain edge of the channel (*V*_i,D_), and *L*_1_ = 26 μm and *L*_2_ = 5 μm to track the intrinsic potential near the source edge (*V*_i,S_). The intrinsic drain and source potentials (*V*_D_ and *V*_S_) are also extracted.

The employed CAD tool constitutes a modular compact modeling framework designed to simulate GFET-based circuits by incorporating the fundamental physical principles governing the device response. It supports analysis across DC, time-domain (transient), and frequency-domain (AC and noise) regimes.^19^ The model has been extensively validated through comprehensive characterization of diverse graphene-based circuits, including voltage amplifiers, high-efficiency frequency doublers, subharmonic mixers, and multiplier phase detectors, demonstrating a strong correlation between experimental measurements and simulation data.^19,43,54,55^ Moreover, the GFET CAD tool has been successfully applied to the design and analysis of ambipolar circuits, such as analog phase shifters, baluns, tunable frequency multipliers, voltage-controlled amplifiers, modulators, and subharmonic mixers,^56–59^ enabling the implementation of novel graphene-based analog architectures.

Although only the core DC module of the CAD tool is employed in this work, we take advantage of one of its most recent updates: the inclusion of asymmetric carrier mobilities,^51^ which allows the definition of distinct mobilities for electrons and holes. As observed in Figure 6a, our fabricated graphene technology exhibits a higher transconductance in the electron-dominated conduction branch (*V*_B_ > *V*_Dirac_).

Table 1 summarizes the physical and geometrical parameters of the GFET that are characterized in Figure 6. The measured and simulated transfer characteristics, as well as the internal potential drops along the channel near the drain and source electrodes, are shown in Figure 8, exhibiting good agreement. Given that the internal electrodes have a width of 2 μm (cf. Figure S5), two independent simulations were performed by adjusting *L*_1_ and *L*_2_ according to the two experiments described in Four Terminal Devices. Specifically, for the experiment measuring *V*_i,D_, *L*_1_ = 4 μm and *L*_2_ = 27 μm were used, whereas for the experiment characterizing *V*_i,S_, *L*_1_, = 26 μm and *L*_2_ = 5 μm were considered (cf. Figure 7). In both cases, the total device length was kept constant at *L* = 31 μm, which differs by approximately 3% from the device length defined in the layout (30 μm). Different electron (μ_n_ = 5500 cm^2^/(V s)) and hole (μ_p_ = 2500 cm^2^/(V s)) mobilities were considered as the transconductance (*g*_m_ = d*I*_DS_/d*V*_B_) exhibited clear asymmetry between the hole-dominated (*V*_B_ < *V*_Dirac_, with *V*_Dirac_ ≈ −26 V) and electron-dominated (*V*_B_ > *V*_Dirac_) conduction branches, as shown in Figure 6. Source and drain contact resistances of *R*_s_ = 490 Ω and *R*_d_ = 550 Ω were included, which are reasonably consistent with the contact resistance values extracted using the adapted Y-function methodology described in Four Terminal Devices and illustrated in Figure 4. As discussed in Analytical Solution of the Potential Distribution Along the Graphene-Based Field-Effect Transistor Channel, due to the presence of non-negligible contact resistances in GFETs, the intrinsic source (*V*_S_) and drain (*V*_D_) potentials at the active channel edges (highlighted in Figure 7) cannot be directly shorted to reference in practice (cf. eq 2). These contact resistances produce a voltage drop; therefore, for the applied *V*_DS,ext_ = 5 V, the corresponding internal *V*_S_ and *V*_D_ potential drops at the graphene channel edges are presented in the Supporting Information, Figure S6.

**Table 1:** Physical and Geometrical Parameters of the Ad Hoc Fabricated Graphene Technology Employed in the GFET CAD Tool Described in refs 19 and 51

parameter	value
*L* [μm]	31
*W* [μm]	20
*C*_b_ [nF/cm^2^]	7.25
*V*_B0_ [V]	–28.2
μ_n_ [cm^2^/s]	5500
*R*_s_ [Ω]	490
*n*_res_ [cm^–2^]	2.9 × 10^11^
μ_p_ [cm^2^/(V s)]	2500
*R*_d_ [Ω]	550

**Figure 8:**
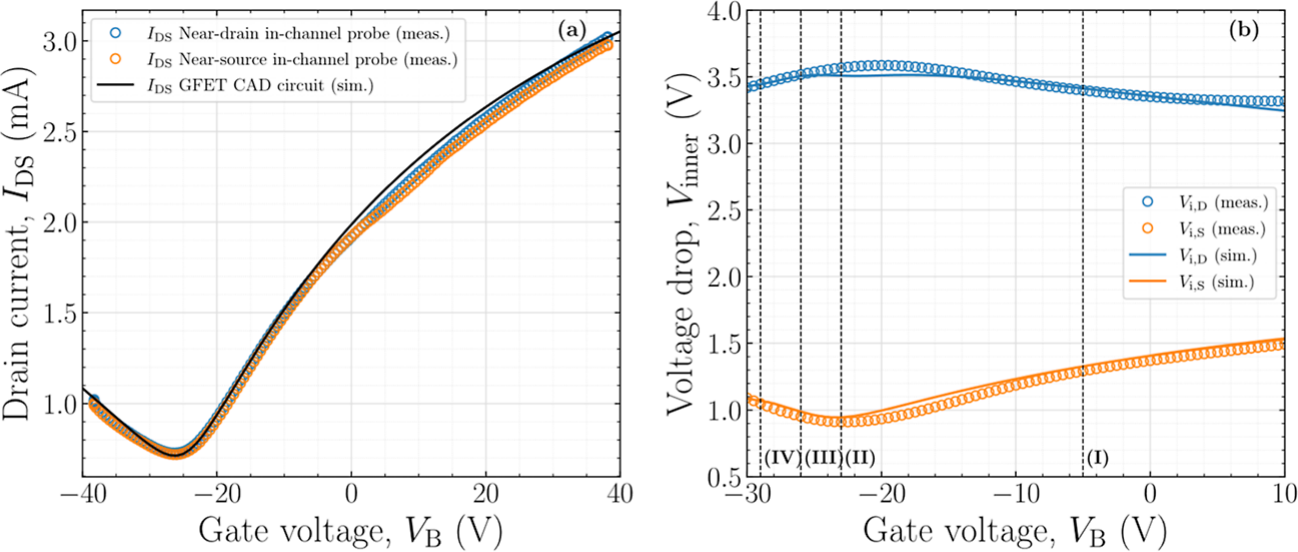
Measured and simulated (a) transfer characteristics and (b) the internal potential drops along the channel, near the drain and source electrodes, of the GFET described in Table 1, for *V*_DS,ext_ = 5 V. Four representative bias points, spanning different electrostatic and transport regimes of the device, are delimited by dashed lines.

In Figure 8b, four representative bias points are identified and selected for subsequent analysis in order to experimentally assess the validity of the analytical models describing the potential distribution along the GFET channel, as introduced in Analytical Solution of the Potential Distribution Along the Graphene-Based Field-Effect Transistor Channel. These operating points span the different transport regimes of the device and are defined as follows: (I) *V*_B_ = −5 V, corresponding to an electron-dominated conduction regime; (II) *V*_B_ = −23 V, which coincides with the bias condition yielding the maximum transconductance, *g*_m_ = 60.3 μS; (III) *V*_B_ = −26 V, corresponding to the Dirac voltage (*V*_Dirac_ = −26 V), where ambipolar transport dominates; and (IV) *V*_B_ = −29 V, associated with a hole-dominated conduction regime.

Figure 9a–d depicts the electrostatic potential distribution along the graphene channel for bias conditions (I)–(IV), respectively, as calculated using Methodologies A and B. In each panel of Figure 9, the voltage drops measured at the inner electrode locations, positioned near the source and drain edges, are explicitly highlighted. This representation enables a direct comparison between the model predictions and the experimentally measured local potentials across the different conduction regimes, demonstrating good agreement between theory and experiment.

**Figure 9:**
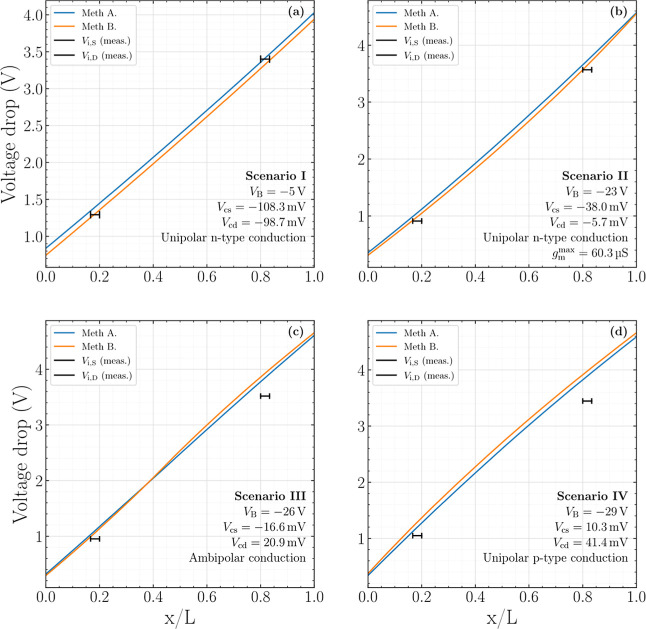
Measured (black solid lines) and predicted potential distributions along the GFET, calculated using Methodology A (blue solid lines) and Methodology B (orange solid lines), for four bias scenarios: (a) Scenario I, *V*_B_ = −5 V; (b) Scenario II, *V*_B_ = −23 V; (c) Scenario III, *V*_B_ = −26 V; and (d) Scenario IV, *V*_B_ = −29 V. The chemical potentials at the source and drain channel edges are also provided. The externally applied drain voltage is *V*_D,ext_ = 5 V.

Additional supporting results are provided in the Supporting Information. In particular, Figure S6 shows the predicted gate-voltage dependence of the chemical potentials at the source and drain channel edges (*V*_cs_ and *V*_cd_, respectively), while Figures S7a–d illustrate the corresponding chemical potential distributions along the graphene channel, calculated using Methodology A, for the four bias scenarios (I)–(IV), thereby complementing the analysis of the potential profiles. The chemical potentials at the graphene channel edges are of paramount importance as they directly govern carrier injection at the metal–graphene contacts and therefore play a key role in understanding and optimizing contact-limited transport in GFETs.^18^

The potential distributions shown in Figure 9 exhibit an approximately linear dependence along the device length, in agreement with previous studies^17^ applied to long-channel GFETs. In this context, similar GFET architectures incorporating a single in-channel electrode were previously proposed in ref 37, where the potential profile of long-channel GFETs was exploited for rectification and signal inversion functionalities. In this regard, Figure S8 presents the benchmarking of the proposed analytical methodology for the potential distribution when applied to the GFET technology reported in ref 37, using the physical and geometrical parameters summarized in Table S1 (Supporting Information).

Figure 9a presents Scenario I (*V*_B_ = −5 V), which corresponds to an electron-dominated (n-type) conduction regime. An excellent agreement is obtained between the experimentally extracted in-channel potentials and the theoretical predictions. Moreover, the two modeling approaches employed, Methodologies A and B, yield similar results in terms of the potential distribution under these bias conditions, confirming the consistency of the proposed framework. As discussed in Analytical Solution of the Potential Distribution Along the Graphene-Based Field-Effect Transistor Channel, the validity of Methodology B relies on the condition |*V*_c_| ≫ *k*_B_ *T*/*q*,^52^ which ensures that the approximated expression for calculating the quantum capacitance of graphene is accurate.^50^ This requirement is fully satisfied in Scenario I, where |*V*_c_| > 3*k*_B_ *T*/*q* along the entire channel, as also evidenced in Figure S7a. In addition, the chemical potential remains negative throughout the graphene channel (*V*_c_ < 0 V), indicating that the Fermi level (*E*_F_ = −*qV*(*x*)) lies above the Dirac energy (*E*_D_ = −*q*(*V*(*x*) – *V*_c_(*x*))) at all positions, i.e., *E*_F_ – *E*_D_ > 0 eV. Consequently, transport is governed by unipolar electron conduction. A closely related behavior is observed in Scenario II (*V*_B_ = −23 V), depicted in Figure 9b, which corresponds to the bias condition yielding the maximum transconductance. As in Scenario I, the agreement between experimental data and simulations remains excellent, and both methodologies provide consistent potential profiles, even though the condition |*V*_c_| ≫ *k*_B_ *T*/*q* is not strictly satisfied along the entire channel length. This behavior can be attributed to the relatively large applied drain voltage (*V*_DS,ext_ = 5 V), for which the electrostatic potential *V* largely exceeds *V*_c_ (cf. Figure S6); however, for bias conditions involving lower *V*_DS,ext_, where *V* and *V*_c_ become comparable, Methodology B is expected to deviate from the results obtained using Methodology A. In this context, the relative error between Methodology B and Methodology A is quantified in Figure S9 (Supporting Information), showing that it remains below 12% for all the considered bias scenarios and below 6% when averaged along the channel length. These results confirm that the deviation introduced by Methodology B remains limited under the analyzed operating conditions. The impact of the quantum capacitance approximation in Methodology B is further discussed in ref 50.

A key difference between Scenarios I and II nevertheless emerges near the drain edge: as shown in Figure 9b and more clearly in Figure S7b, the chemical potential at the drain edge approaches zero (*V*_cd_ → 0 V). This indicates the onset of channel pinch-off at the drain side. This condition has been widely identified as the bias point at which the transconductance reaches its maximum value, and it also coincides with the onset of quasi-current saturation in the output characteristics (*I*_DS_ – *V*_DS_).^17,43,60–62^ In this regime, the output conductance *g*_ds_ = d*I*_DS_/d*V*_DS_ reaches a minimum, which is particularly advantageous for high-frequency operation and for maximizing the intrinsic voltage gain, *A*_i,v_ = *g*_m_/*g*_ds_.^63–65^ The present results therefore provide a clear electrostatic interpretation of the optimal bias condition for performance of multiple applications based on graphene-based FETs, such as high-frequency, sensor, and related analog circuits.

In contrast, Scenario III (*V*_B_ = −26 V) corresponds to an ambipolar transport regime and coincides with the Dirac voltage (*V*_Dirac_) observed in the transfer characteristics shown in Figure 8a. The ambipolar nature of transport is directly confirmed by the opposite signs of the chemical potentials at the channel edges: *V*_cs_ > 0 V at the source edge and *V*_cd_ < 0 V at the drain edge. This indicates electron conduction near the source (Fermi level above the Dirac energy, i.e., *E*_F_(*x* = 0) – *E*_D_(*x* = 0) > 0 eV) and hole conduction near the drain (Fermi level below the Dirac energy, i.e., *E*_F_(*x* = *L*) – *E*_D_(*x* = *L*) < 0 eV). Furthermore, as widely reported in the literature,^17,25,61^ the minimum conduction condition in GFETs is achieved when the Fermi level aligns with the Dirac energy at the center of the channel. Our results are fully consistent with this picture as *V*_c_ ≈ 0 V is obtained at *x*/*L* ≈ 0.44, as shown in Figure S7c. An analysis of the impact of gate electrostatics on the ambipolar potential profile is provided in Figure S10 (Supporting Information), showing that thinner oxides lead to sharper transitions around the channel midpoint.

Finally, Scenario IV (*V*_B_ = −29 V), as shown in Figure 9d, corresponds to a hole-dominated (p-type) conduction regime. In this case, both the source and drain chemical potentials satisfy *V*_cs_ > 0 V and *V*_cd_ > 0 V, indicating that the Fermi level remains below the Dirac energy along the entire graphene channel (cf. Figure S7d). While the electrostatic potential distribution itself is independent of the drain current and carrier mobility, as discussed in Analytical Solution of the Potential Distribution Along the Graphene-Based Field-Effect Transistor Channel, the intrinsic boundary potentials at the source and drain (*V*_S_ and *V*_D_, respectively) are computed by explicitly accounting for the voltage drops across the metal–graphene contact resistances (eq 2). As a consequence, the pronounced asymmetry between electron and hole mobilities of our technology is expected to affect the quantitative agreement between simulated and experimentally extracted in-channel potentials under p-type dominated operation. This effect likely explains the slightly increased discrepancies observed in hole-dominated conduction, as shown in Figure 9c,d.

Taking all these results into consideration, both the self-consistent and explicit analytical models of the potential distributions in a GFET channel (Methodologies A and B, respectively) have been successfully validated against experimental data across the full range of operating regimes. These models therefore constitute pioneering analytical tools for accurately describing in-channel potential profiles in GFETs. The proposed methodologies provide valuable physical insight into the different regimes of operation of GFETs, including unipolar electron and hole transport, ambipolar conduction, and onset of channel pinch-off. As such, they enable a clear electrostatic interpretation of key device features that are directly linked to performance metrics of practical relevance. Importantly, the analytical nature and computational efficiency of the proposed models make them especially suitable for device design and optimization, facilitating rapid exploration of the bias conditions and geometrical parameters. This capability is particularly advantageous for the development of GFETs targeting high-frequency electronics, sensing platforms, and other analog circuit applications, where a detailed understanding of the internal potential distribution is essential for achieving optimal performance.

## Conclusions

In this work, a pioneering set of self-consistent and explicit closed-form analytical expressions has been developed to compute the potential distributions in GFETs. The proposed models have been experimentally validated using a custom-fabricated test platform based on global back-gated GFETs incorporating two in-channel terminals, which enable direct measurement of the potential drop at different positions along the graphene channel. This experimental capability provides a unique means of probing the internal electrostatics of GFETs.

The fabricated GFET technology has been comprehensively characterized, including a detailed extraction of metal–graphene contact resistances for different test structure footprints using a methodology that explicitly accounts for the underlying charge transport physics. Measured drain currents and in-channel potential profiles have been systematically compared to physics-based simulations, demonstrating excellent agreement across all investigated bias conditions.

Both the self-consistent and explicit analytical methodologies have been validated over a wide range of operating regimes, encompassing unipolar n-type and p-type conductions, ambipolar transport, and the bias condition corresponding to maximum transconductance. The extracted potential distributions further enable a clear physical interpretation of key transport phenomena in GFETs, including the onset of channel pinch-off associated with maximum transconductance, the role of channel electrostatics in carrier injection from contacts, and the intrinsic unipolar or ambipolar nature of graphene transport under different biasing conditions.

The analytical framework presented in this work provides a powerful and computationally efficient tool for the rapid evaluation, optimization, and interpretation of the GFET experimental results. Beyond conventional analogue electronic applications, this framework is directly applicable to emerging fields such as graphene-based sensing, where local electrostatic potentials govern sensitivity and selectivity to chemical and biological analytes; flexible electronics, where the interplay between mechanical deformation, electrostatics, and charge transport must be efficiently captured; and thermoelectric applications, where spatial potential gradients play a central role. As such, the proposed models offer a versatile and insightful foundation for the design and analysis of next-generation graphene-based electronic systems.

## Supplementary Material



## Data Availability

The data sets generated and/or analyzed during the current study are available from the corresponding author on reasonable request.

## Notes

Additional SEM images and layout diagrams; contact resistance extraction plots for additional channel widths; layout of the four-terminal graphene-based FET, featuring two in-channel electrodes (i,S) and (i,D), with the relevant geometrical dimensions highlighted; chemical potential plotted against channel percentage and gate voltage; relative error between Methodologies A and B for the predicted potential distributions along the channel position; and model for the drain-source current (PDF)
