# Supplementary material for: Analytical Solution for the Potential Distribution in the Channel of A Graphene Field-Effect Transistor Validated with a Custom-Fabricated Test Platform
Source: ACS Appl Electron Mater. 2026 Apr 30;8(9):4213–25. doi: 10.1021/acsaelm.6c00360 (PMC13173535; doi:10.1021/acsaelm.6c00360)
Supplement: Supplementary file 1 [file el6c00360_si_001.pdf]

Supporting Information

Analytical solution for the potential distribution in the channel of a graphene field-effect transistor validated with a custom-fabricated test platform

Antonio Cantudo[1], Francisco Pasadas[*1,2], Anibal Pacheco-Sánchez[1,2], Rem Elnahas[3,4], Miguel Muñoz Rojo[4], Juan Bautista Roldán[*1,5]

Address:
[1]Departamento de Electrónica y Tecnología de Computadores, Facultad de Ciencias, Universidad de Granada, 18071 Granada, Spain.
[2]Pervasive Electronics Advanced Research Laboratory (PEARL), Facultad de Ciencias, Universidad de Granada, 18071 Granada, Spain.
[3]Catalytic Processes and Materials Group, Faculty of Science and Technology, MESA+ Institute for Nanotechnology, University of Twente, PO Box 217, 7500 AE Enschede, The Netherlands.
[4]Thermal Engineering group, Faculty of Engineering Technology, University of Twente, PO Box 217, 7500 AE Enschede, The Netherlands.
[5]Institute "Carlos I" for Theoretical and Computational Physics, University of Granada, 18071, Granada, Spain

Corresponding author email: jroldan@ugr.es, fpasadas@ugr.es

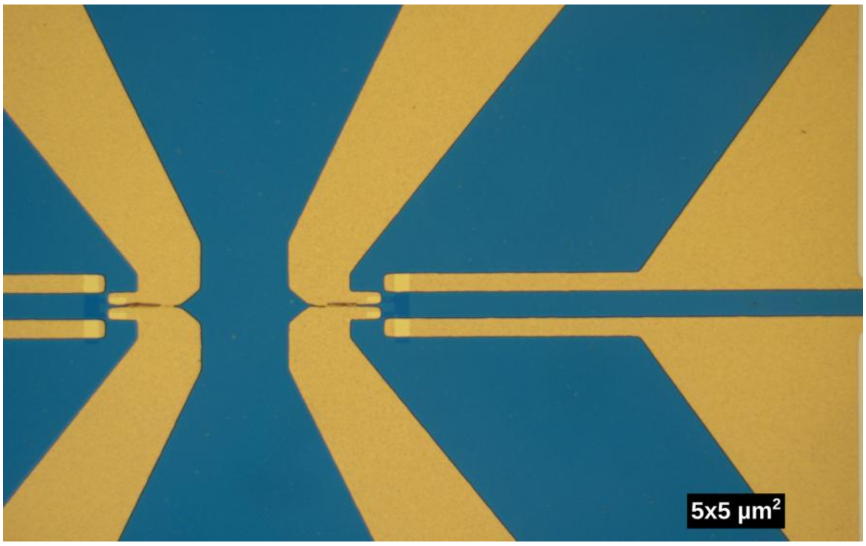

Figure S1. SEM image of the fabricated GFET structures showing the four terminals contacting the graphene channel. From the image, some mask misalignment issues are observed.

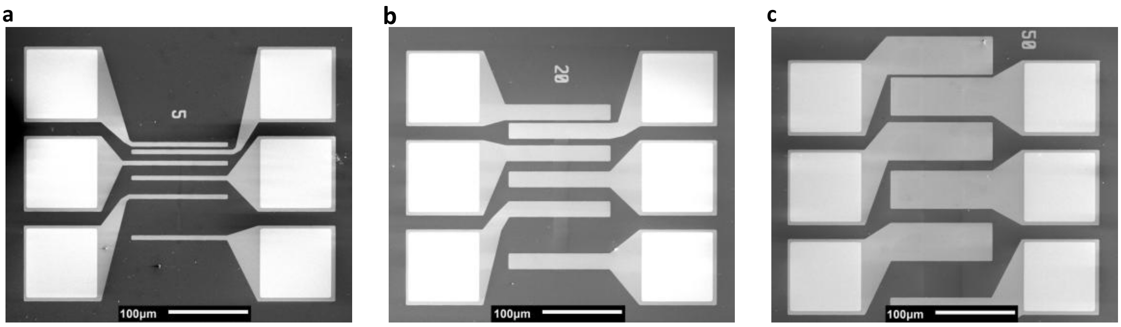

Figure S2. SEM images of some of the measured GFET structures for three different channel widths, **a** 5 μm, **b** 20 μm, and **c** 50 μm**.**

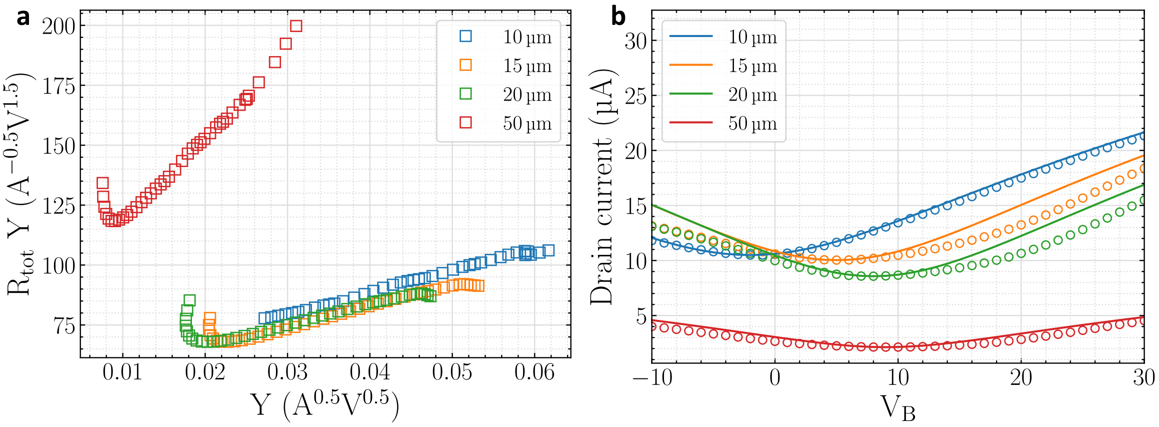

Figure S3. **a** $R_{tot}Y$ versus Y function plot for GFETs with channel width of 15 μm and different channel lengths. **b** Experimental (symbols) and modeled (solid lines, Supporting Note 1) transfer characteristics for GFETs with channel width of 15 μm and different channel lengths.

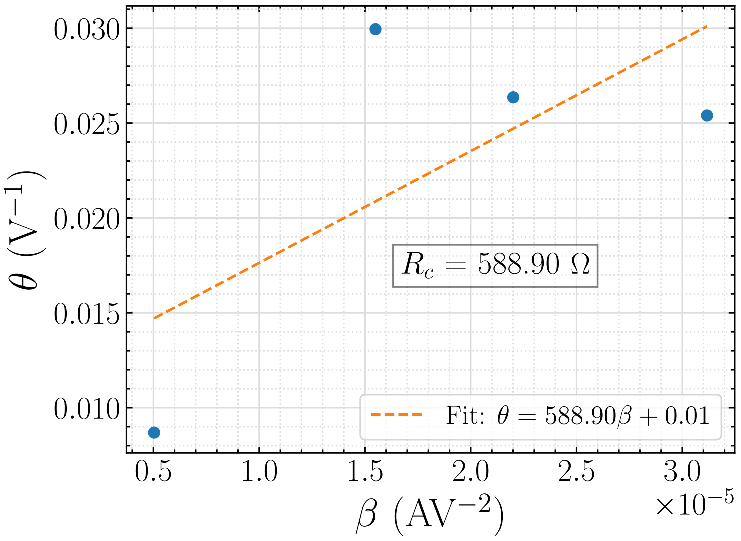

Figure S4. θ parameter versus β. Experimental data are shown with symbols, and the dashed line represents the modeling results[1] by using the extracted parameters.

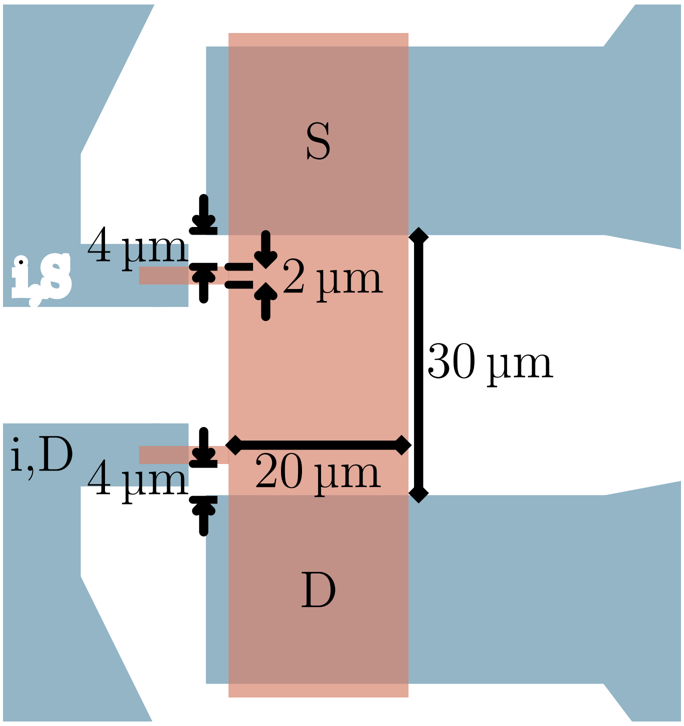

Figure S5. Layout of the proposed four-terminal graphene-based FET, featuring two in-channel electrodes (i,S) and (i,D), with the relevant geometrical dimensions highlighted.

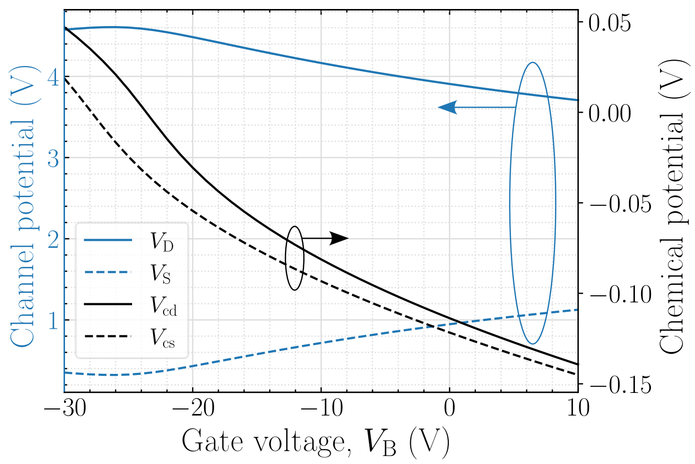

Figure S6. Gate-voltage dependence of the channel potentials (blue) and chemical potentials (black) at the drain (solid lines) and source (dashed lines) edges of the graphene channel. The externally applied drain voltage is $V_{D,ext}$ = 5V.

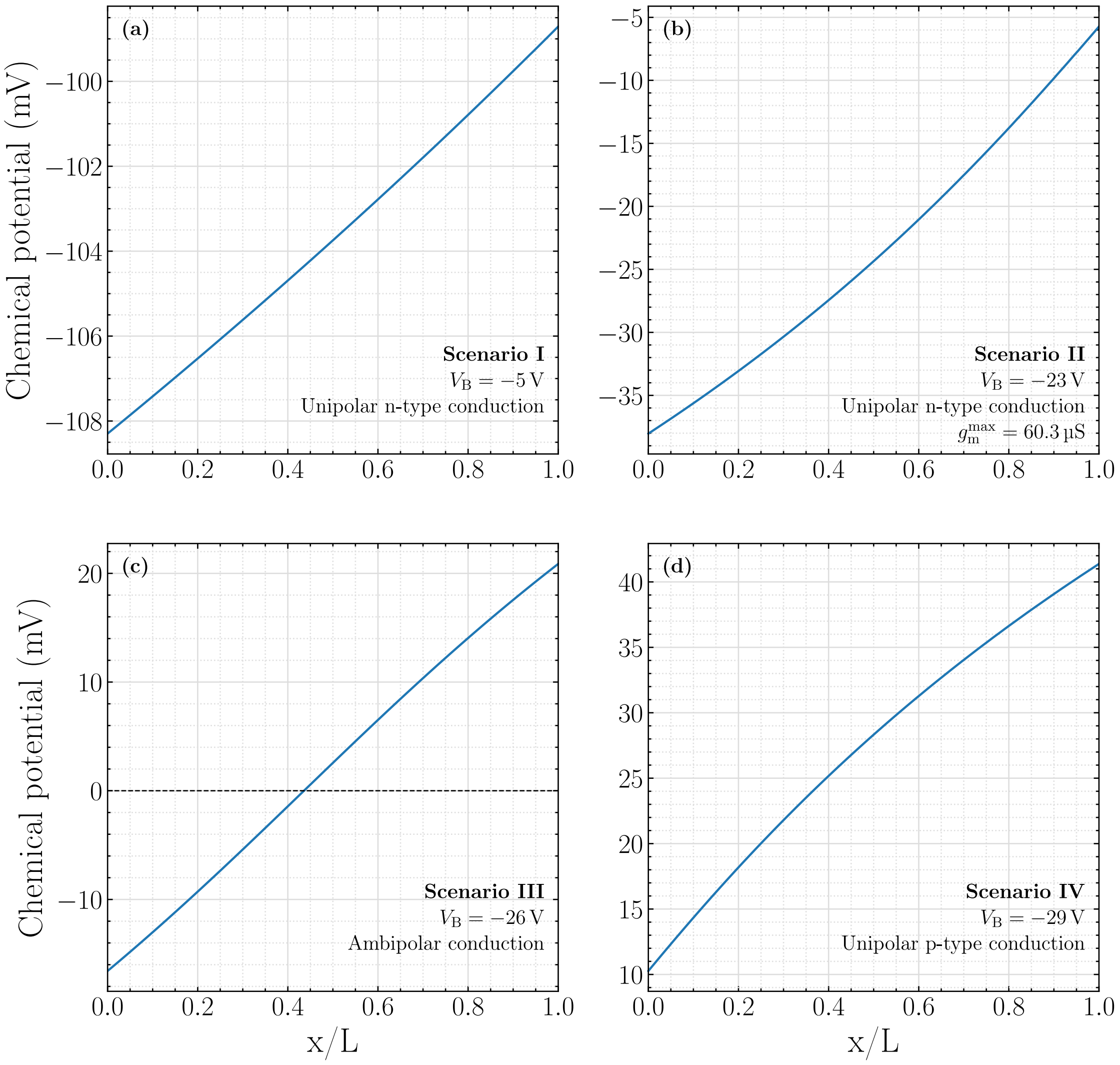

Figure S7. Predicted chemical potential distributions along the GFET, calculated using Methodology A, for four bias scenarios: **a** Scenario I, V_B = -5V; **b** Scenario II, V_B = -23V; **c** Scenario III, V_B = -26V; and **d** Scenario IV, V_B = -29V. The externally applied drain voltage is $V_{D,ext}$ = 5V.

Table S1: Physical and geometrical parameters employed in the GFET CAD tool[2] for describing the electrical performance of the graphene technology presented in ref. 3.

Parameter	Value	Parameter	Value	Parameter	Value
L [μm]	28	V_{G0} [V]	-0.5	n_{res} [cm^{-2}]	4.4×10^{11}
W [μm]	54	μ_n [cm^2/s]	2250	μ_p [cm^2/Vs]	2250
C_t [nF/cm^2]	266.8	R_s [Ω]	300	R_d [Ω]	300

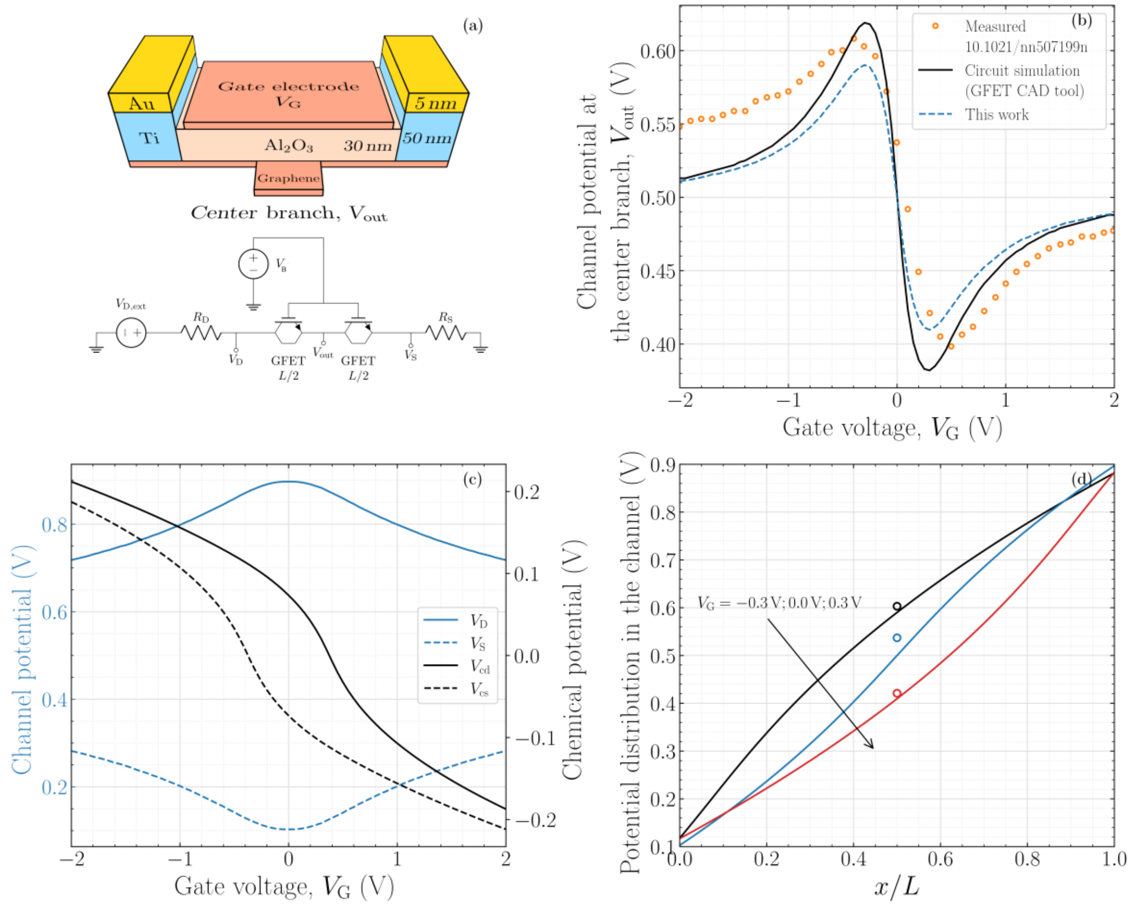

Figure S8. GFET-based inverter reported in ref. 3. (a) Schematic cross-section of the device (reprinted with permission from ref. 3. Copyright 2015 American Chemical Society) and corresponding circuit topology, where the device is modeled as two cascaded GFETs with half of the original channel length ($L/2$ = 14 μm), with V_{out} taken at the central contact. (b) Measured (symbols), circuit-simulated (solid line), and analytically predicted in-channel potential at the channel midpoint (V_{out}), obtained using Methodology A, as a function of gate voltage for $V_{DS,ext}$= 1 V. (c) Gate-voltage dependence of the channel potentials (blue) and chemical potentials (black) at the drain (solid lines) and source (dashed lines) edges of the graphene channel ($V_{DS,ext}$= 1 V). (d) Measured (symbols) and analytically predicted potential distributions along the GFET, calculated using Methodology A, for three representative bias conditions (V_G = −0.3 V, 0 V, and 0.3 V) at $V_{DS,ext}$= 1 V.

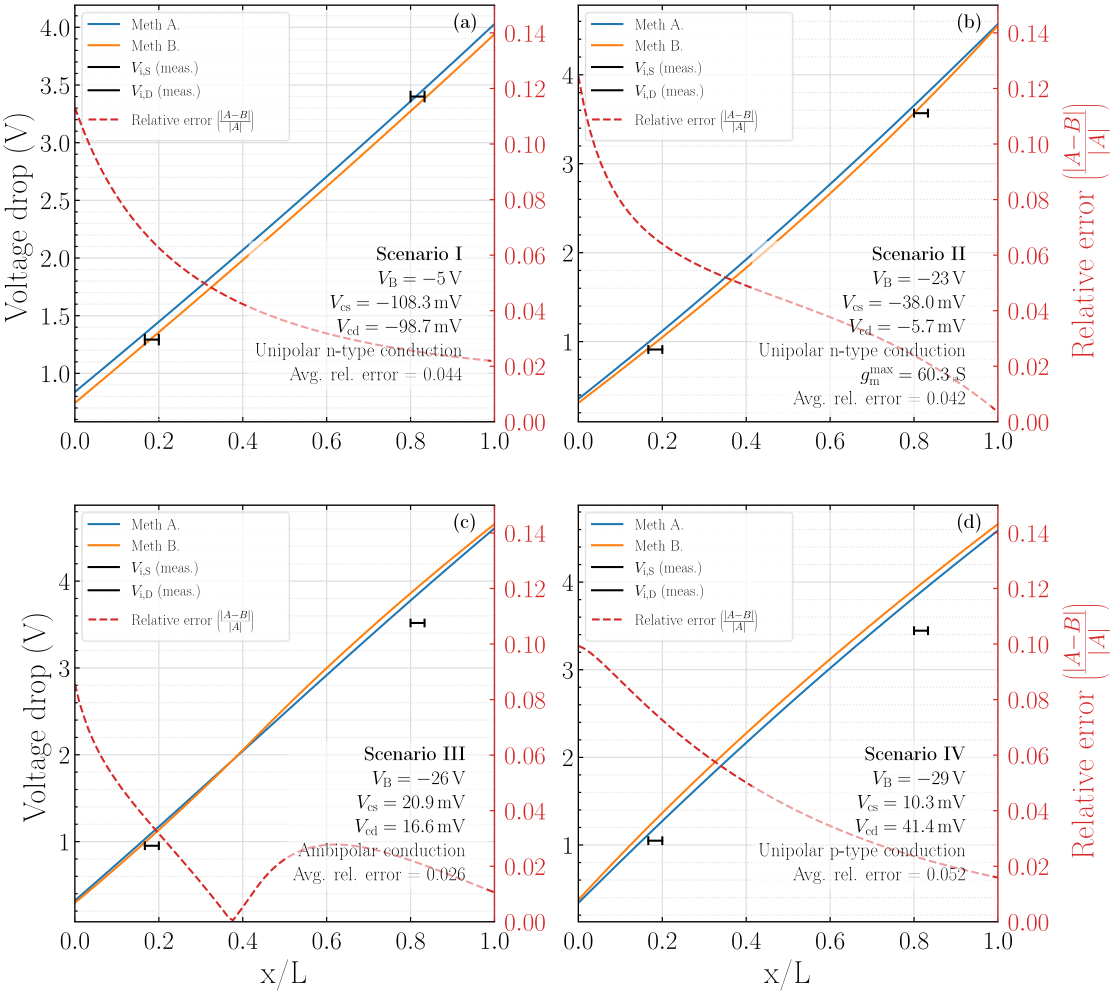

Figure S9.- Measured (black solid lines) and predicted potential distributions along the GFET channel using Methodology A (blue solid lines) and Methodology B (orange solid lines), together with the relative error (red dashed line, right axis), for four bias scenarios: a Scenario I, V_B = -5V; b Scenario II, V_B = -23V; c Scenario III, V_B = -26V; and d Scenario IV, V_B = -29V. The chemical potentials at the source and drain channel edges are also indicated, along with the average relative error in each case. The externally applied drain voltage is $V_{D,ext}$ = 5V.

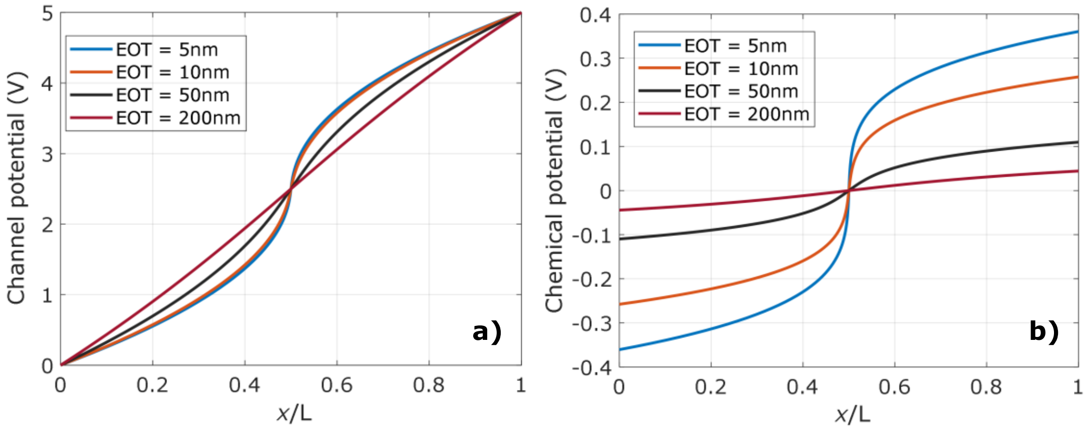

Figure S10.- Spatial distributions of **a)** channel potential and **b)** chemical potential along the graphene channel for different equivalent oxide thickness (EOT) values ranging from 5 nm to 200 nm. The back-gate voltage is set at the Dirac voltage ($V_{\mathrm{B}} = (V_D + V_S)/2$),[4] with $V(x = 0) = V_{\mathrm{S}} = 0$ V and $V(x = L) = V_{\mathrm{D}} = 5$ V. The results illustrate the impact of gate electrostatics on the ambipolar potential profile, with thinner oxides leading to sharper transitions around the channel midpoint.

Supporting Note 1: $I_{DS,Y}$ model equation, used for experimental transfer curve fitting through contact resistance extraction[1]:

$$I_{\mathrm{DS,Y}} = \beta \frac{\sqrt{V_0^2 + V_{\mathrm{BCO}}^2}}{1 + \theta\sqrt{V_0^2 + V_{\mathrm{BCO}}^2}} V_{\mathrm{DS,ext}} \qquad \text{(S1)}$$

where $V_0 = q \times n_{res}/C_b$ is the residual voltage with q as the electron charge, n_{res} as the residual charge carrier density at V_{Dirac}, and C_b the geometrical back-gate dielectric capacitance per unit area.

References

[1] A. Pacheco-Sanchez, N. Mavredakis, P. C. Feijoo, D. Jiménez, "An Extraction Method for Mobility Degradation and Contact Resistance of Graphene Transistors," in IEEE Transactions on Electron Devices, 69, 4037-4041, 2022. https://doi.org/10.1109/TED.2022.3176830.

[2] F. Pasadas, P. C. Feijoo, N. Mavredakis, A. Pacheco-Sanchez, F. Chaves, D. Jiménez, "Compact Modeling Technology for the Simulation of Integrated Circuits Based on Graphene Field-Effect Transistors," in Advanced Materials, 34 (48), 2022. https://doi.org/10.1002/adma.202201691.

[3] W. Kim, C. Li, N. Chekurov, S. Arpiainen, D. Akinwande, H. Lipsanen, J. Riikonen, "All-Graphene Three-Terminal-Junction Field-Effect Devices as Rectifiers and Inverters," in ACS Nano 9 (6), 5666–5674, 2015. https://doi.org/10.1021/nn507199n.

[4] F. Pasadas, A. Medina-Rull, P. C. Feijoo, A. Pacheco-Sanchez, E. G. Marin, F. G. Ruiz, N. Rodriguez, A. Godoy, D. Jiménez, "Unveiling the impact of the bias-dependent charge neutrality point on graphene based multi-transistor applications," in Nano Express, 2, 036001, 2021. https://doi.org/10.1088/2632-959X/abfdd0.
